# Acetazolamide: Old drug, new evidence?

**DOI:** 10.1002/epi4.12619

**Published:** 2022-06-14

**Authors:** Arif Ali Shukralla, Emma Dolan, Norman Delanty

**Affiliations:** ^1^ The National Epilepsy Programme Beaumont Hospital Dublin Ireland; ^2^ FutureNeuro, The SFI Research Centre for Chronic and Rare Neurological Disease Dublin Ireland; ^3^ Royal College of Surgeons in Ireland Dublin Ireland

**Keywords:** acetazolamide, acid‐sensing ion channel, carbonic anhydrase inhibitor, systematic review

## Abstract

Acetazolamide is an old drug used as an antiepileptic agent, amongst other indications. The drug is seldom used, primarily due to perceived poor efficacy and adverse events. Acetazolamide acts as a noncompetitive inhibitor of carbonic anhydrase, of which there are several subtypes in humans. Acetazolamide causes an acidification of the intracellular and extracellular environments activating acid‐sensing ion channels, and these may account for the anti‐seizure effects of acetazolamide. Other potential mechanisms are modulation of neuroinflammation and attenuation of high‐frequency oscillations. The overall effect increases the seizure threshold in critical structures such as the hippocampus. The evidence for its clinical efficacy was from 12 observational studies of 941 patients. The 50% responder rate was 49%, 20% of patients were rendered seizure‐free, and 30% were noted to have had at least one adverse event. We conclude that the evidence from several observational studies may overestimate efficacy because they lack a comparator; hence, this drug would need further randomized placebo‐controlled trials to assess effectiveness and harm.

**Key points**

- Acetazolamide is a carbonic anhydrase inhibitor used to treat epilepsy.
- Its mechanism of action involves acidification and its effects on acid‐sensing ion channels (ASICs), and this has a facilitatory effect on other receptors such as NMDA, GABAa and glycine.
- The evidence for the efficacy and safety of acetazolamide is from older studies, with an overall responder rate of 49%.
- Acetazolamide is a safe and effective adjunct as an anti‐seizure medication.

## 1 INTRODUCTION

Epilepsy is commonly treated with anti‐seizure medications (ASMs), of which a plethora of choices now exist. In 1940, Mann and Kellin discovered Acetazolamide and recognized its anticonvulsant property against focal and generalized seizures.^1^ Acetazolamide is available in tablet form. The typical dose ranges from 250 to 4000 mg/d and is administered in two to four divided doses; its half‐life is 6‐8 hours, and it is eliminated in the urine. Acetazolamide is marketed as *Diamox*. Despite its potential anti‐seizure effects, acetazolamide is rarely used due to perceived adverse events and the development of tolerance. In this review, we discuss the mechanism of action of acetazolamide and review the current clinical evidence for its use.

## 2 METHODS

We used MEDLINE to search for clinical trials of acetazolamide in epilepsy using the search terms: acetazolamide, epilepsy, seizures, and clinical trials. We also searched for studies of acetazolamide and its mechanism of action. Trials were analyzed systematically and, where possible, using meta‐analysis; we used narrative reviews as appropriate. We first discuss the mechanism of action of acetazolamide and review the evidence of efficacy and harm.

### 2.1 Mechanism of action

Acetazolamide is a noncompetitive inhibitor of carbonic anhydrase containing a sulpha ring moiety that plays a vital role in the mechanism of action.^2^ Other carbonic anhydrase inhibitors such as zonisamide, topiramate, and sulthiame also possess a sulpha ring. Acetazolamide is a weak diuretic, but the anticonvulsant mechanism of action is not related to this.^3^ Velíšek et al. demonstrated that carbonic anhydrase knockout mice are less likely to have provoked seizures due to low plasma bicarbonate^4^; therefore, they hypothesized that the anticonvulsant action of acetazolamide is due to a process of acidification, and understanding how carbonic anhydrase maintains pH is important. First, we shall discuss carbonic anhydrases and how acetazolamide affects these. Next, we can discuss the role of pH in neuronal networks and how acid‐sensing ion channels (ASICs) are important.

### 2.2 Carbonic anhydrases

Carbonic anhydrases (CA) are zinc metalloenzymes ubiquitous in nature and found in several human tissues.^5^ These enzymes are prevalent in the cytosol, mitochondria, and the cell surface membrane. Cells that express CA include neurones, oligodendrocytes, astrocytes, and choroid plexus cells, where they regulate CFS production, maintain pH and extracellular space or play a role in cell signaling.^6^ Humans have 16 different isoforms of carbonic anhydrase, named numerically; isoform two is concentrated in the choroid plexus, where it regulates CSF production, and isoforms 4, 5, 7 and 12 are found in the hippocampus.^5^, ^6^

Carbonic anhydrases catalyze the conversion of carbon dioxide and water to bicarbonate and hydrogen ions in a reversible multistep process.^5^, ^7^ The catalytic site of all carbonic anhydrases is highly conserved; it is composed of a half hydrophobic and hydrophilic site with a zinc ion, held in place by three histidine residues, one water molecule, and a hydroxide ion.^8^

The equation is as shown: CO_2_ + H_2_O = HCO3^−^ + H^+^. First, the hydroxide bound zinc ion in the active site binds and fuses with carbon dioxide to form an intermediate ion. Next, the intermediate ion reversibly binds to water to produce a bicarbonate‐zinc‐water complex, which later expels a proton (H^+^) to reform the bound zinc hydroxide and free bicarbonate.^5^

Carbonic anhydrase inhibition by acetazolamide results in a reduction in the concentration of free bicarbonate ions; this, in turn, reduces the buffering capacity of cells internally, resulting in an acidic intracellular pH. Carbonic anhydrases are also located on the cell surface, influencing extracellular pH. Furthermore, in the kidneys, acetazolamide promotes bicarbonate secretion in the proximal tubule, resulting in alkaline urine and metabolic acidosis hence contributing to the systemic effects of acetazolamide.^9^, ^10^ It is beyond dispute that acetazolamide causes acidification; what is needed is a discussion on how this affects neurons.

### 2.3 Effects of a low pH on neuronal function

Acetazolamide induced pH changes are crucial to its anti‐seizure effect. Evidence from a magnetic resonance spectroscopy study in humans showed that in the interictal state, the side of the seizure focus is more alkaline compared to the contralateral side, with a pH level of 7.25 on the ipsilateral side vs 7.08 on the contralateral side, indicating that a change of about 0.2 may be sufficient to either protect against seizure or cause seizures.^11^ Animal models too have shown that a pH change of 0.2 is sufficient to alter excitability. A pH of 6.8 has been shown to protect against seizure propagation, whereas a pH of 7.7 is proconvulsant.^12^, ^13^

Protons are secreted alongside neurotransmitters and play a role in the modulation of cell signaling.^14^ Protons can affect NMDA receptors; an alkaline pH with zinc can cause NMDA receptors to undergo conformational change and remain open for extended periods. Alkalosis in the CA3 cells of the hippocampus increases the epileptic potential of the cell by increasing glutamate activity; therefore, acidification increases the seizure threshold.^15^, ^16^

Seizures can be affected by pH, temperature changes, and hyperventilation. These are generalized seizures, including febrile seizures, absence seizures, neonatal seizures, and status epilepticus. Febrile seizures, for example, are caused by hyperventilation caused by temperature changes, which leads to alkalosis, which affects neuronal excitability. Furthermore, hyperventilation can cause vasoconstriction, which contributes to a proconvulsant state. Hyperventilation in the absence of fever can also induce EEG changes in susceptible children, which can be attenuated by carbon dioxide supplementation.^17^, ^18^ Hyperventilation is also known to occur in Rett syndrome; a mouse model of Rett syndrome showed that hyperventilation causes alkalosis, which made the CA1 neurones of the hippocampus hyperexcitable at a pH of 8.4.^19^

Carbogen is a mixture of 5% carbon dioxide and oxygen and was thought to have an act as an anticonvulsant; inhalation of carbogen in a rat model caused a decrease in onset latency of seizures and reduced frequency of seizures with a corresponding reduction in glutamate levels and an increase in GABA levels.^20^ However, two studies using carbogen in humans did not show any potential benefit; the first was an EEG study in patients with nonconvulsive status epilepticus (NCSE).^21^ The second study by Forsyth et al. 2016 did not show any significant effect in pediatric patients with NCSE.^22^

Seizures are common in newborns, and most of these are due to birth asphyxia. The current hypothesis is that birth asphyxia leads to neuronal alkalosis initially due to acid excretion by the blood–brain barrier. Animal models evidence for this by Helmy et al. showed that after asphyxia in rats, the pH in the brain rises, and there is a net efflux of acid from the brain. Moreover, inhibition of the sodium proton channels causes suppression of seizures.^23^

We have demonstrated how acidosis acts to alleviate or prevent seizures, which provides indirect evidence of the effect of acetazolamide. Additional evidence that acidosis has an anti‐seizure effect is based on many mechanisms. These can be summarized as follows: extracellular acidosis inhibits NMDA receptors, either limiting or stopping seizures from propagating; extracellular acidosis also increases adenosine levels that interact with adenosine receptors, thereby having an anti‐seizure effect. Finally, extracellular acidosis modulates GABAa receptors, enhancing the inhibitory effect of seizures.

### 2.4 The role of acid‐sensing ion channels in epilepsy

In the past decade, we have learned more about the structure and function of ASICs and their role in epilepsy. Understanding how these channels work may provide some insight into how acetazolamide works. There are four main types of ASICs, and these are highly conserved across species. ASIC1a is important in the central nervous system. ASIC1a is found in the cortex, limbic structures, and piriform cortex.^24^, ^25^ ASICs are found in neurons and astrocytes with a role in cell signaling; other cells such as oligodendrocytes and microglia also express these in smaller amounts.^26^

ASICs comprise an extracellular domain with an acid‐sensing pocket and a transmembrane portion that allows sodium and calcium to pass.^26^ Acid‐sensing ion channels are activated at different pH ranges; for example, ASIC1a is activated at a pH of 6.8, ACIS1a/2b is activated at pH 4.8‐5.4, and ASIC3 is activated at a pH of 6.6. Pathological states such as seizures, status epilepticus, and ischemia reduce the extracellular pH near 7.35‐6.8, where ASICs are activated.

ASICs' role is controversial; it is not clear whether they serve to protect the brain against seizures or are part of the ictogenesis pathway. However, what is clear from several studies is that ASICs may account for the link between pH changes and seizures. First, activation of ASICs causes small depolarisation; this, however, is not sufficient in strength to cause seizure propagation, so it is thought that the depolarisation has a facilitatory effect on other receptors such as NMDA, GABA, and Glycine.^26^, ^27^, ^28^ Second, ASICs are expressed in some neurons more than others; they are expressed in higher densities in interneurons which are predominantly inhibitory, and they are expressed on astrocytes where they too have an inhibitory role.^29^ This was confirmed by a study using hippocampal neurones that showed that ASICs have a role in GABAergic inhibition rather than depolarisation.^30^ GABAergic interneurons have more ASICs than glutamate, resulting in a GABAergic inhibition when pH becomes more acidic.^27^ Two studies found that some interneurons express more ASICs than others and are more prone to ischemic injury, and their loss due to injury makes the network proconvulsant; their results confirm that acidosis decreases seizure susceptibility via ASICs.^27^, ^31^ Similarly, another study found that activated astrocytes show enhanced ASIC levels, which may contribute to epileptogenesis in a TLE mouse model.^32^

In conjunction with ASICs, bicarbonate and chloride transporters also regulate pH. These are either sodium channel‐dependent or sodium independent. These transporters are prominent in astrocytes, where they absorb acid from the environment or release acid into the extracellular space. Furthermore, Theparambil et al.^33^ demonstrated that a change to an acidic pH is enough to inhibit NMDA and voltage‐gated calcium channels, thereby decreasing the propensity to seizures. Not only did their study show the well‐known effect of pH, but astrocytes will act as a buffer when the environment becomes too acidic or alkaline. Hence, all these point to a mechanism whereby acidosis achieves its anti‐seizure effect.^33^

### 2.5 Acetazolamide and how it works

**FIGURE 1:**
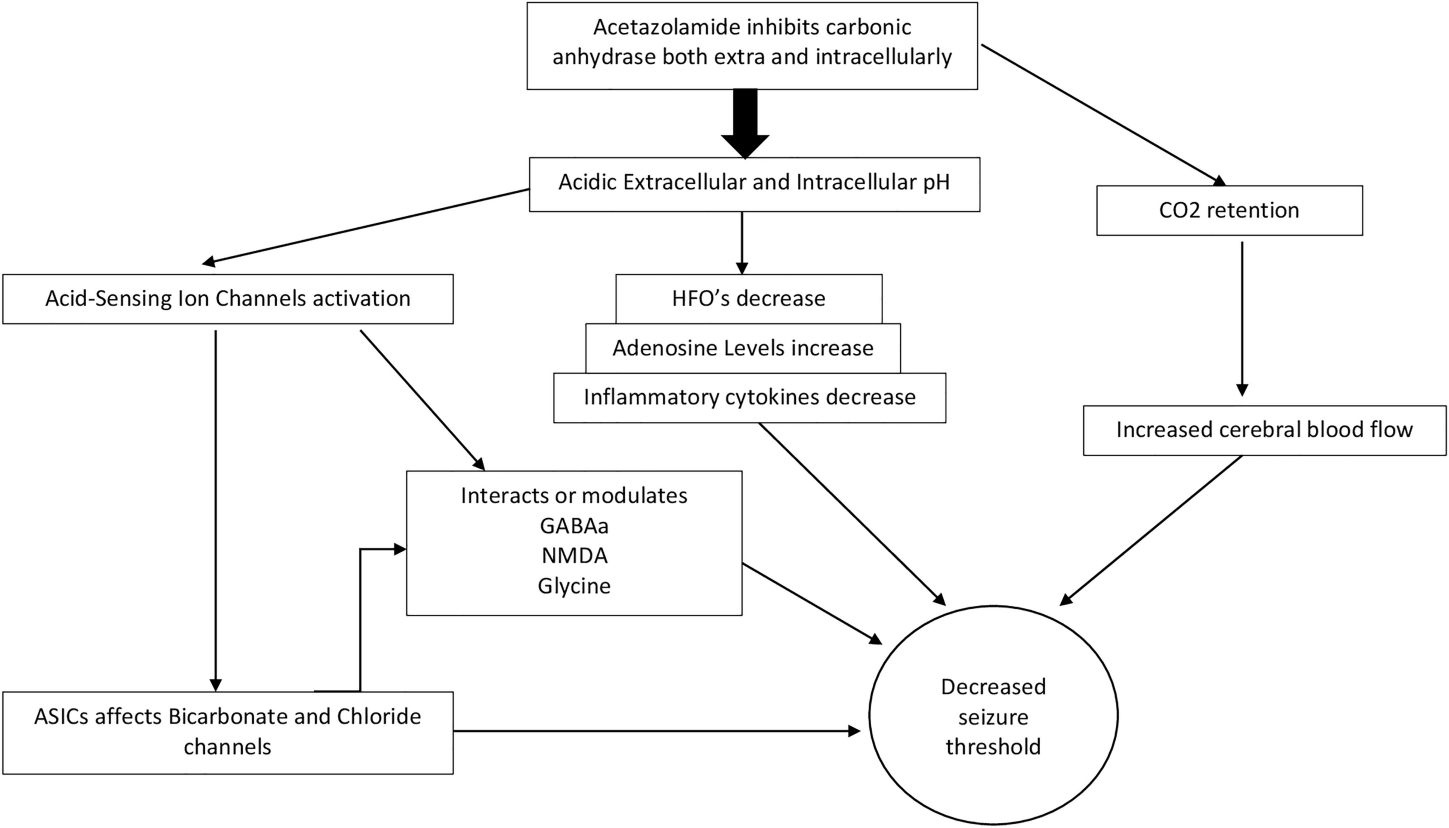
This diagram summarizes the critical mechanism of action of acetazolamide. By inhibiting carbonic anhydrase, acetazolamide inhibits the conversion of CO_2_ and water to bicarbonate and protons, increasing the pH in both intracellular and extracellular environments. In addition to CO_2_ retention and increased blood flow, the acidic pH affects ASICs and modulates several receptors. These, in turn, decrease the seizure threshold of groups of neurons in the hippocampus, entorhinal cortex and piriform cortex

These studies indirectly point to how acetazolamide by acidification poses a therapeutic effect, but in conjunction with these other evidence, may also add other ways whereby acetazolamide interacts with the central nervous system; a summary of these is found in Figure 1.

High‐frequency oscillations (HFOs) are essential in interictal discharges in patients with epilepsy; HFOs are biomarkers of refractory epilepsy and are thought to occur in the epileptogenic zones of patients with both focal epilepsy and non‐focal epilepsy^34^; HFOs also have a role in identifying the seizure onset zone in epilepsy surgery. GABAergic interneurons are involved in the generation of HFOs.^35^ Work by Hamidi and Avoli showed that acetazolamide reduced the duration of ictal discharges in the piriform cortex and entorhinal cortex, decreased the occurrence of HFOs, and decreased the amplitude and duration of HFOs.^36^ Changes in internal pH also affect synaptic vesicle transport, a lowering of pH by acetazolamide alters vesicle recycling across the synapse in rodent models, thereby reducing the release of acetylcholine.^37^

Evidence from single neuron studies showed that both sulthiame and acetazolamide reduce the pH of CA3 hippocampal neurones in a guinea pig model of epilepsy, thereby decreasing the epileptogenic potential of these cells to produce ictal discharges even when the carbonic anhydrase inhibitor was washed out of the cell.^38^ Finally, a further mechanism of action may be due to the retention of carbon dioxide, resulting in increased cerebral blood flow, thereby increasing the seizure threshold. This mechanism has not been proven in humans, but data exists in animal models.^39^ These models explain some of the ways seizures are affected by pH. The effect of acetazolamide does this by influencing the Bohr effect by preventing the offloading of oxygen in tissues, and this is compensated by an increase in cerebral blood flow.^40^

Epilepsy is associated with increased brain inflammatory cytokines, and seizures induce these increases. These, in turn, activate nuclear transcription of NF‐kB, complement and chemokines, which result in inflammation in the brain. Brain inflammation is proconvulsive, and some purport that antiepileptic drugs may have a role as an anti‐inflammatory effect. During neuronal injury, ASICs are activated, and these activate inflammasomes leading to neuronal injury and blockage of ASICs will reduce this injury.^41^ Acetazolamide decreases the levels of inflammatory cytokines IL‐6, TNF alpha and IL‐1beta in rat models of epilepsy. Acetazolamide also reduces inflammation by reducing cytokine expression, further contributing to its antiepileptic effect.^41^, ^42^

Although the exact mechanism of how acetazolamide interacts with sodium channels is unknown, there is indirect evidence that it may play a role here. Severe myoclonic epilepsy of infancy (SMEI) or Dravet syndrome is a rare disorder causing seizures and encephalopathy. Mutations of SCN1A are responsible for 80% of cases of SMEI. To validate an SCN1A rat model, Ohmori et al. used clinical seizures and ictal electroencephalography to test various ASMs, including acetazolamide. They found that when mutant rats experienced temperatures of 45 degrees, they were induced to have febrile seizures. This may be due to thermal hyperpnea, which is known to be proconvulsant in addition to SCN1A mutation effects.^43^ The model demonstrated findings contrary to what one might expect; both acetazolamide and carbamazepine decreased the temperature threshold where seizures would occur, suggesting that they are pro‐convulsive in this setting; again, this may be due to acetazolamide acting as a respiratory stimulant.^44^ Despite this, ictal electrographic recordings showed that the duration of seizures was decreased with acetazolamide, whereas carbamazepine did not reduce the seizure duration. A motor coordination test, which is a test of ataxia in these rats, showed acetazolamide did not impair this, whereas carbamazepine did cause ataxia.^43^ One can conclude that in a rat model of Dravet syndrome, acetazolamide does not help prevent seizures but helps abolish seizures when they do occur. Nevertheless, the clinical role of acetazolamide in Dravet syndrome has not been reported.

### 2.6 Clinical studies of acetazolamide

Several trials of acetazolamide have evaluated it to treat altitude sickness and idiopathic intracranial hypertension; however, the evidence for using acetazolamide in epilepsy is sparse. We carried out a search strategy for clinical trials using Medline. We searched for the terms “epilepsy” and “acetazolamide.” A total of 248 abstracts were found; 47 were not epilepsy articles; 65 were scientific reports; 48 were review articles; 21 were case reports; 42 were not in English; two were letters, and 11 were not available electronically. A total of 12 articles were therefore included. A summary of the selected studies is shown in Table 1, and the efficacy outcomes, adverse events, and risk of bias are shown in Table 2.

**TABLE 1:** Characteristics of studies included

Study	Type of study	Number of patients	Age range of patients	Duration of treatment	Included patients	Acetazolamide dose	Schedule	Outcomes measures	Adverse events reported
Ansell and Clarke (1956)^45^	Prospective Single‐arm trial	26	Children and adults	12 to 30 mo	Patients with idiopathic 23 and symptomatic epilepsy 3 not responded to other AEDs	250 mg/d Add‐on	Add‐on	Efficacy described as % of patients with “excellent,” “good,” “some value,” and “no value” EEG changes to the treatment	Yes
Lombroso et al (1956)^52^	Prospective Single‐arm trial	150	Children and adults	Not stated	Patients with idiopathic and symptomatic epilepsy not responded to other AEDs or new patients with epilepsy	250 mg to 1500 mg/d	Add‐on and Monotherapy	Efficacy described as % of patients with “100% control,” “>90% control,” “> 50% control” and “<50% control” EEG changes to the treatment	Yes
Millichap (1956)^53^	Prospective double arm with placebo	14	Children	5–26 wk	Patients with idiopathic or symptomatic epilepsy with abnormal EEG changes who had failed several AEDs	18–36 mg/kg/d	Add‐on	Percentage of seizure control form baseline	Yes
Holowach and Thurston (1958)^48^	Prospective single‐arm trial	56	Children	Not stated	Patients with idiopathic or symptomatic epilepsy failed other AEDs	<10–90 mg/kg/d	Add‐on	Efficacy described as % of patients with Group A‐ complete control; group B >50% reduction of seizures; group C “little value”	Yes
Ross (1958)^55^	Non‐randomized prospective placebo‐controlled trial	70	Children and adults	Not stated	Patients with idiopathic or symptomatic epilepsy failed other AEDs	250–750 mg/d (63 patients 7 patients given a placebo	Add‐on and monotherapy	Efficacy described as % of patients with “Prolonged effect,” “Temporary effect,” and “no effect”	Yes
Lombroso and Forxythe (1959)^51^	Prospective single‐arm trial	257	Children and adults	3 y	Patients with idiopathic or symptomatic epilepsy failed other AEDs or new patients with epilepsy	250‐1500 mg/d	Add‐on and monotherapy	Efficacy described as % of patients with “100% control,” “>90% control,” “>50% control” and “<50% control” at 3 mo, at 12 mo at 2 and 3 y. Subgroup by seizure type EEG changes to the treatment	Yes
Wada et al. (1961)^56^	Prospective single‐arm trial	36	Children and adults	Up to 4 y	Patients with possible idiopathic or symptom epilepsy. Characteristics of patients not fully stated as such	750 mg Monotherapy or add‐on	Add‐on and Monotherapy	Efficacy described as % of patients with >90%; between 90% and 50%; between 50% and 10%; unchanged seizure frequency: pts with exacerbated seizures	Not reported
Chao and Plumb (1961)^46^	Retrospective review	178	Children	2 mo to 3 y	Patients with possible symptomatic epilepsy or idiopathic as the description were not clear	15‐30 mg/kg/d Monotherapy or Add‐on	Add‐on and Monotherapy	Efficacy described as % of patients with 80%‐100% control; 50–80% control, <50% control; worsening of seizures EEG changes to acetazolamide	Yes
Forsythe et al. (1981)^47^	Prospective single‐arm trial	54	Children	Up to 5 y	Patients with either idiopathic or symptomatic epilepsy that found no benefit from carbamazepine	10‐15 mg/kg/d	Add‐on	The proportion of patients seizure‐free at 2, 3, 4 and 5 y	Yes
Oles et al. (1989)^54^	Retrospective review	48	Children and adults	Up to 30 mo	Patients with focal epilepsy who did not benefit from carbamazepine or other AEDs	3.8–16.5 mg/kg/d	Add‐on	The proportion of patients who had a reduction of seizures >505 of baseline over a 3‐mo period	Yes
Iype et al. (2000)^49^	Prospective single‐arm study	15	Adults	Up to 30 mo	Patients with focal epilepsy on either carbamazepine plus primidone or phenytoin plus primidone	10 mg/kg/d	Add‐on	The proportion of patients with a reduction in seizure frequency compared to baseline. The reported absolute reduction in seizure frequency as a percentage	Yes
Katayama et al. 2002^50^	Prospective single‐arm study	37	Children	Up to 200 wk	Patients with focal epilepsy and generalized epilepsy where acetazolamide was used as an add‐on	10‐20 mg/kg/d	Add‐on	Efficacy outcomes not reported a priori but reports the proportion of patients seizure‐free, the proportion of patients with >50% reduction n seizures frequency Drug levels of acetazolamide	Yes

**TABLE 2:** Efficacy outcomes, adverse events and risk of bias of included studies

Study	Efficacy results (%)	The proportion of patients with a cumulative 50% or greater in a reduction of seizures frequency at any point in time (%)	Seizure types	The proportion of patients seizure‐free (%)	Number of patients with at least one adverse event (%)	Adverse events % (no)	Risk of bias
Ansell and Clarke (1956)^45^	26 patients included, 26 patients evaluable 8 patients “Excellent outcome” 6 patients “Good outcome” 8 patients “Some Value” 4 patients “No value”	No estimate computed	Focal to bilateral tonic–clonic Focal with impaired awareness Generalized tonic clonic	No estimate computed	5 (19%)	Paraesthesia Drowsiness Depression	No random sequence allocation, No Allocation concealment and no blinding of the outcome. Possible outcome reporting bias
Lombroso et al. (1956)^52^	150 patients included, 126 evaluable 34 patients sz free 12 patients >90% reduction in sz frequency 21 patients >50% reduction in sz frequency 58 patients <50% reduction in sz frequency	68/126 (54%)	Generalized tonic clonic Myoclonic	34/126 (26%)	59 (39%)	Drowsiness 19 Anorexia 17 Irritability 11	No random sequence allocation, No Allocation concealment and no blinding of the outcome. Possible outcome reporting bias No outcome reported for 24 patients
Millichap (1956)^53^	14 patients included, 14 evaluable 6 patients >90% reduction in seizure frequency 11 patients >50% reduction in seizure frequency 3 patients <50% reduction in seizure frequency	11/14 (79%)	Generalized tonic clonic Myoclonic Possibly focal seizures as well	3/14 (21%)	10 (71%)	Anorexia 5 Polyuria 5 Nocturnal enuresis 4 Drowsiness 3 Pallor 2 Vomiting 1 Diarrhea 1 Two patients withdrew treatment due to AE	No random sequence allocation No allocation concealment No blinding of outcome measurement
Holowach and Thurston (1958)^48^	56 patients included, 56 evaluable 35 patients seizure‐free 9 patients >50% reduction in seizure frequency 12 patients with “little value”	44/56 (79%)	Focal to bilateral tonic–clonic Focal with impaired awareness Generalized tonic clonic	35/56 (63%)	7 (13%)	7 patients in total had adverse events Lethargy 2 Drowsiness 2 Paraesthesia 2 Increased seizures 1 Natural enuresis 1	No random sequence allocation, No Allocation concealment and no blinding of the outcome. Possible outcome reporting bias
Ross (1958)^55^	73 patients included, 66+7patients evaluable 2 patients “Prolonged effect” 2 patients “temporary effect” 59 patients no effect 7 patients given placebo with no change in seizure frequency	No estimate computed	Focal to bilateral tonic clonic seizures Focal with impaired awareness	No estimate computed	5 (71%)	Nausea Dizziness Tingling in extremities Glare	No random sequence allocation, No Allocation concealment and no blinding of the outcome. Possible outcome reporting bias Although a placebo was present as comparator
Lombroso and Forxythe (1959)^51^	257 patients included an 257 patients evaluable 19 patients with 99 to 100% control 23 patients with 90 to 99% control 17 patients with >50% seizure control 41 patients with <50% seizure control 19 patients could be considered seizure‐free	59/257 (23%)	Generalized tonic clonic Possibly Focal seizures	19/257 (7%)	61 (24%)	Drowsiness Anorexia Irritability Nausea Vomiting Enuresis Paraesthesia Headache Thirst Dizziness Hyperventilation	No random sequence allocation, No Allocation concealment and no blinding of the outcome. Possible outcome reporting bias
Wada et al. (1961)^56^	36 patients included and 36 patients evaluable 14 patients >90% reduction in sz frequency 6 patients with 50% to 90% control 4 patients with 50% to 10% control 10 patients with <10% control	20/36 (56%)	Generalized tonic clonic Myoclonic Possibly focal seizures	No estimate computed	Not reported	Not reported	No random sequence allocation, No Allocation concealment and no blinding of the outcome. Possible outcome reporting bias Incomplete outcomes
Chao and Plumb (1961)^46^	178 patients included and 178 evaluable 76 patients with >80% Sz reduction 44 patients between 50 to 80% sz reduction 44 patients <50% reduction 3 patients worsened seizures	120/178 (65%)	Focal to bilateral tonic clonic seizures Focal with impaired awareness Generalized tonic clonic	No estimate computed as no data on how many patients seizure‐free	Total Number of patients not reported	Anorexia 27 Drowsiness 11 Vomiting 11 Irritability 8 Headache 7 Fatigue 6 Dizziness 5 Enuresis 5 Paraesthesia 4 Ataxia 3 Depression 3 Irregular respiration 2 Polyuria 2 Poor sleep 2 Abdominal distension 1 Cyanosis 1	No random sequence allocation, No Allocation concealment and no blinding of the outcome. Possible outcome reporting bias
Forsythe et al. (1981)^47^	54 patients included and 54 evaluable The proportion of pts with seizures at 3 mo 1, 2, 4 and 5 y 16 patients reported being seizure‐free	No estimate computed as selection bias exists in selecting a time to include	Focal to bilateral tonic clonic Focal with impaired awareness Generalized tonic clonic	16/54 (29%)	10 (18%)	Drowsiness 8 Ataxia Nausea and vomiting 1	No random sequence allocation, No Allocation concealment and no blinding of the outcome. Possible outcome reporting bias
Oles et al. (1989)^54^	48 patients include and 48 patients evaluable 12 patients with >50% r reduction in sz frequency 9 patients >75% reduction in seizure frequency 4 patients with >90% reduction in seizure frequency 3 patients seizure‐free	25/48 (52%)	Focal to bilateral tonic clonic Focal with impaired awareness Generalized tonic clonic	3/48 (6%)	10 (21%)	3 patients withdrawn due to AE Lethargy 4 Paraesthesia 6 Anorexia 2 Headache 1 Nausea 3 Diarrhea 2 Visual changes 1	No random sequence allocation, No Allocation concealment and no blinding of the outcome. Possible outcome reporting bias
Iype et al. (2000)^49^	15 patients included and 15 evaluable 8 patients seizure‐free 11 patients had >50% reduction in seizure frequency 2 patients had <50% reduction in seizure frequency 2 patients had worsened seizures	11/15 (73%)	Focal to bilateral tonic clonic Focal with impaired awareness	8/15 (53%)	1 (7%)	Paraesthesia 1	No random sequence allocation, No Allocation concealment and no blinding of the outcome. Possible outcome reporting bias
Katayama et al. (2002)^50^	37 patients included and 37 evaluable 4 patients seizure‐free 6 patients with >50% reduction in seizure frequency	10/37 (27%)	Focal to bilateral tonic clonic Focal with impaired awareness Generalized tonic clonic Myoclonic	4/37 (11%)	28 (76%)	Not reported	No random sequence allocation, No Allocation concealment and no blinding of the outcome. Possible outcome reporting bias

### 2.7 Characteristics of studies

A total of 12 studies were included in this review representing 941 patients with epilepsy.^45^, ^46^, ^47^, ^48^, ^49^, ^50^, ^51^, ^52^, ^53^, ^54^, ^55^, ^56^ The ages of patients were mixed; one study exclusively recruited adults, five studies exclusively children, and six studies both adults and children. Study duration varied widely, but all were long, up to 2 years. Two studies by Iype et al. and Oles et al. recruited patients with focal epilepsy only; all the other studies included patients with both focal and generalized epilepsy.^49^, ^54^

Ten studies were prospective, and two studies by Oles et al., Chao & Plumb were retrospective.^46^, ^54^ The studies were all observational studies (Table 1). These seizures were a mix of generalized tonic–clonic seizures, focal to bilateral tonic–clonic, focal with impaired awareness seizures or myoclonic seizures (Table 2). Five studies recruited children only, with patients having both focal and generalized seizures. Only Iype et al^49^ recruited adults with focal seizures exclusively. Therefore, the evidence of acetazolamide presented here will apply to both focal epilepsy and generalized epilepsy syndromes (Table 1).

One study had placebo as a comparator,^55^ where seven patients were given a placebo, but this was a non‐randomized study, and details of treatment allocation were not given, and the study was not blinded. Another study also administered a placebo, but this was not comparative group patients were allocated this at baseline.^53^ Three studies evaluated acetazolamide where carbamazepine had failed, and six studies evaluated acetazolamide where other ASMs, including carbamazepine, had failed. Wada et al^56^ 1961 did not mention if their patients had failed other ASMs, but they were given acetazolamide as an add‐on or monotherapy. Chao & Plumb^46^ did not indicate what prior ASMs their patients were given. Seven studies used acetazolamide as add‐on therapy, and six studies used acetazolamide as monotherapy in some patients or polytherapy in others (Table 1).

Dosages of acetazolamide varied widely across trials making comparisons between studies difficult as some reported total doses per day; others reported dose per weight per day. Only two trials reported patients taking large doses of acetazolamide up to 1500 mg/d.^51^, ^52^

Efficacy outcomes varied across all the studies, making direct comparisons difficult. Four studies included EEG changes as part of the efficacy outcome. Ansell & Clark described efficacy using words such as “excellent,” “good,” or of “some value.” Holowach & Thurston categorized patients as 100% controlled, >50% controlled or of little value.^45^, ^48^ Ross used other descriptive terms such as “prolonged effect,” “temporary effect,” or “no effect”.^55^ Six studies used a percentage threshold to describe outcomes. Iype et al. reported an absolute value of the reduction of seizure for each patient in his cohort.^49^ Despite the heterogeneity in outcome reporting, one can still estimate the proportion of patients whose seizure frequency was reduced by >50%. It was possible in some studies to deduce the proportion of patients seizure‐free.

Due to the varied methodology, population characteristics, variable dose and schedules, and lack of a control group, it was impossible to perform a meta‐analysis due to significant clinical and statistical heterogeneity. Here, we describe the outcomes narratively.

### 2.8 Efficacy outcomes, responder rate

Despite the variation in outcomes in the studies, we calculated the responder rate in some studies. This is shown in Table 2. The responder rate was calculated in nine studies. Three studies could not have their responder rate calculated as the outcomes were poorly reported; thus, the total number of patients in nine studies was 767. The percentage of patients with more than 50% reduction in seizures ranged from 23% reported in Lombroso & Forxythe to 79% in Millichap, Holowach & Thurston.^48^, ^51^, ^53^ There is little correlation between the dose of acetazolamide, or methodology to account for any differences in the responder rate magnitude. A total of 368 patients of 767 had a >50% reduction in seizure frequency, yielding a mean responder rate of 48%. A total of 821 patients were assessed for any benefit from acetazolamide, and 567 patients met that outcome, yielding a percentage of 66%.

### 2.9 Seizure freedom

Eight of 12 studies reported the proportion of patients seizure‐free after treatment with acetazolamide, thus providing data on 607 patients. Seizure freedom rates varied from 6% in the Oles et al. study to 63% in the Holowach & Thurston study.^48^, ^54^ Significant heterogeneity in seizure freedom rates is due to methodological differences and reporting bias. A total of 122 of 607 patients were seizure‐free, giving a mean seizure freedom rate of 20%.

### 2.10 Adverse events

Harms outcomes were reported in 11 of the 12 studies. One study did not provide any harms outcomes^56^ and the second study by Chao & Plumb reported proportions of patients with adverse events.^46^ However, the study did not provide a total number of patients reporting harms. Therefore, 10 studies reported the number and proportions of patients reporting adverse effects harms; 196 patients had at least one adverse event out of 664 patients yielding an overall harms rate of 30%. Proportions of patients reporting side effects ranged from 7% in one study to 76% in another. The most frequently reported adverse events included paraesthesia followed by drowsiness, nausea, and dizziness. Paraesthesia occurred in 13 patients reported by Holowach & Thurston 1958; Chao and plumb; Oles 1989 and Iype 2000.^46^, ^48^, ^49^, ^54^ A total of 40 patients reported drowsiness in four studies. Five patients in Chao and Plumb reported dizziness. Chao and Plumb^46^ reported the most significant number of adverse events by category, with 16 discrete adverse events reported. Withdrawals due to adverse events were reported by Oles et al. and Millichap; three patients withdrew due to adverse events in the former, and two patients withdrew due to adverse events in the latter.

### 2.11 Risk of bias

All but one study was placebo‐controlled without allocation concealment, and there was no randomized patient selection. Clinical heterogeneity between studies did not allow for the calculation of a summary statistic. For example, outcome reporting was heterogeneous in Lombroso et al., where 150 patients were included in the study, but the outcomes of only 126 patients were reported.^52^ Some studies did not report harm, and some provided incomplete data for efficacy and safety outcomes.

## 3 DISCUSSION

Acetazolamide is a noncompetitive inhibitor of carbonic anhydrase. Its key mechanism of action is via acidification of the internal milieu, which leads to changes in the activity of neurons and glial cells. Key structures such as the hippocampus are rich in carbonic anhydrase; in addition, ASICs in the hippocampus and cortex have a proven role in epilepsy when neurones respond to changes in pH. Acid‐sensing ion channels have been shown to respond to pH changes, which, in turn, alters neuronal activity by affecting other neurotransmitters and receptors such as NMDA and GABA. Earlier studies of acetazolamide showed that the effect of acetazolamide is not a peripheral effect but a central one. Further studies need to be carried out to develop new small molecules for the treatment of epilepsy. In particular, the interaction with ASICs and acetazolamide may be fruitful in developing newer ASMs.

The evidence of the efficacy and safety of acetazolamide is based on observational studies. These have suggested that there are indeed some benefits of acetazolamide in epilepsy. We reviewed 12 observational studies and found that despite the significant heterogeneity and methodology, acetazolamide is effective, but there are adverse events. Effect sizes vary widely across studies, as observational studies show significant heterogeneity in study design.^57^ Compared to RCTs, observational studies generally tend to report larger effect size. Based on the studies evaluated, one cannot comment on the minimum effective dose, as many studies did not stratify outcomes based on dose. Based on this review, it is unknown how acetazolamide is compared to other ASMs or placebo.

Metabolic acidosis is responsible for most of the side effects of acetazolamide. Acidosis manifests as tiredness, fatigue, and gastrointestinal irritation. The most common side effects are paraesthesia and dizziness. Paraesthesia is reported in 48%‐66% of patients. There is a risk of renal stones, and this can likely be lessened by avoiding a combination of acetazolamide with the other carbonic anhydrase inhibitors, particularly topiramate and zonisamide. In a recent study of children with epilepsy taking topiramate vs acetazolamide and other ASMs, no renal stones were reported in the acetazolamide group.^58^ Concomitant ASMs with carbonic anhydrase inhibitors may pose a risk of hyperammonemia; such ASMs include phenytoin, phenobarbitone, topiramate and zonisamide.^59^ Acetazolamide can, in rare situations, cause hepatotoxicity, which occurs a few days after initiation.

The development of tolerance is an important issue; an increasingly larger dose of acetazolamide is needed to have the same effect. The tolerance mechanism is first due to the increased production of carbonic anhydrase. The second mechanism is via changes in phosphorylation of the enzyme and alterations in synaptic transmission with long‐term use.^60^, ^61^

Acetazolamide has no toxic effects on mitochondrial function; however, it may impair calcium storage in mitochondria by altering cytosolic pH; nevertheless, acetazolamide appears safe in mitochondrial epilepsy.^7^ Allergic and idiosyncratic reactions to acetazolamide are rare.

Several case reports indicate that acetazolamide is unsafe in pregnancy, although one observational study showed contrary evidence. Data from the large pregnancy registries do not have adequate prospective data on the risk of acetazolamide in pregnancy. Reported malformations include acrodactaly, syndactyly, axial skeletal malformations, anophthalmia, microphthalmia, cleft lip and palate and abnormal incisor teeth.^62^ Despite these case reports, a study by Falardeau et al^63^ prospectively reviewed malformations in 101 pregnant women with idiopathic intracranial hypertension taking acetazolamide. These women were taking acetazolamide in the first 13 weeks of pregnancy. The review concluded that the risk of miscarriage was 21% in women not exposed to acetazolamide and 28% in women exposed to acetazolamide. The risk of malformation was also not statistically different. Despite these conflicting findings, acetazolamide is not recommended for women with epilepsy of childbearing potential due to its potential teratogenic effects.

Acetazolamide may have a role in precision therapy of some genetic epilepsies. For example, it may be effective in patients with episodic ataxia and migraine, where pathogenic variants in CACNA1A gene cause these conditions in the CACNA1A. Case reports of patients with mutations in this gene have reported symptoms of episodic ataxia, migraine and epilepsy, with an improvement of symptoms when treated with acetazolamide. In one patient, symptoms of epilepsy, ataxia, and migraine were all abolished with acetazolamide when all other ASMs had failed.^64^, ^65^ The exact mechanism of its presumed targeted interaction with the CACNA1A calcium channel is unknown.^64^

An interesting patient with intractable epilepsy with glaucoma was treated with acetazolamide and dramatically resolved her seizures. In this case, acetazolamide was an adjunct to her other ASMs.^66^ Acetazolamide is also sometimes used off‐license for the treatment of catamenial epilepsy. Acetazolamide is a nonhormonal medication that can be used either perimenstrual as an add‐on to prevent seizures.^67^

Electrical Status Epileptics in Slow Wave Sleep (ESES) is an electrographic phenomenon that typically occurs in Landau–Kleffner syndrome and Continuous Spike and wave in Slow‐wave sleep. These two syndromes are found in children and generally are pharmaco‐resistant to ASMs. A recent case series by Fine et al^68^ described six children with either of these two conditions responding to acetazolamide as an alternative to sulthiame. Three children had complete resolution of symptoms, and five had significant improvement in seizure frequency and other outcome measures such as attention and schooling. BECTS is also treated with sulthiame, and patients with BECTS also have ESES, so by logical conclusion, acetazolamide may also be a good ASM for BECTS. A trial by Pisani et al^69^ evaluated the use of acetazolamide in ESES in children who could not receive sulthiame. Their results showed that all 25 children responded positively with acetazolamide.

## 4 CONCLUSIONS

We conclude that acetazolamide is effective in the treatment of epilepsy and it is safe. This data are from studies that predate the CONSORT method of reporting trials. Acetazolamide works by acidifying the internal milieu in the CNS, thereby affecting neuronal firing through multiple mechanisms of which ASICs are important and should be the focus of new research. Acetazolamide is one of several drugs that use carbonic anhydrase inhibition as its mechanism of action. Clinicians should consider acetazolamide as an adjunct in patients with both focal and idiopathic generalized epilepsy. Novel carbonic anhydrase inhibitors are under development and may shed light on the role of ASICs in epilepsy.

## CONFLICT OF INTEREST

Neither of the authors has any conflicts of interest to disclose. We confirm that we have read the Journal’s position on issues involved in ethical publication and affirm that this report is consistent with those guidelines.
